# Methicillin-resistant *Staphylococcus aureus* contamination and distribution in patient’s care environment at Muhimbili National Hospital, Dar es Salaam-Tanzania

**DOI:** 10.1186/s13104-018-3602-4

**Published:** 2018-07-17

**Authors:** Emmanuel James Nkuwi, Fatima Kabanangi, Agricola Joachim, Sima Rugarabamu, Mtebe Majigo

**Affiliations:** 1 Department of Microbiology and Immunology, College of Health and Allied Sciences, University of Dodoma, Box 259, Dodoma, Tanzania; 2 Department of Microbiology and Immunology, Muhimbili University of Health and Allied Sciences, Box 65001, Dar es Salaam, Tanzania

**Keywords:** MRSA, Patient care environment, Methicillin resistant, Contamination

## Abstract

**Objective:**

Environmental contamination with methicillin-resistant *Staphylococcus aureus* in routine medical care settings poses an increased risk of health care associated infections through cross-transmission. This study aimed at determining the magnitude and distribution of methicillin-resistant *S. aureus* contamination among various items in patients’ care surroundings at Muhimbili National Hospital, Tanzania’s largest tertiary hospital.

**Results:**

A total of 200 environmental samples from high touch items were processed and out of these methicillin-resistant *S. aureus* was 19.5% with significantly higher contamination in general wards. Patients’ beds surfaces were the most contaminated among studied items (43.7%), whilst the surgical trolleys were least contaminated (7.7%). Presence of 10 or more patients in a room was an important significant correlate for methicillin-resistant *S. aureus* contamination by bivariate logistic regression model (odds ratio: 4.75, 95% confidence interval 1.624–13.895, *p* = 0.004). These findings warrant further study of decontamination practices and improved infection control mechanisms, especially in light of the drug resistant isolates identified.

## Introduction

Methicillin-resistant *Staphylococcus aureus* (MRSA) is a leading cause of nosocomial infections in resource-limited countries [1–3]. Globally, MRSA has increasingly became a problem in health care facilities as well as communities, but with greater concern in former settings [4]. Reports from developing countries, including Tanzania have shown as higher as more than 30% of isolates from cases of hospital acquired infections (HAI’s) being MRSA [2]. HAI’s caused by MRSA affect patient care by increasing morbidity, mortality, and costs derived from increased durations of hospitalization and use of more-expensive antimicrobial agents.

There has been mounting evidence that MRSA can be recovered from surfaces and items confined to hospital environments often with increased risk of nosocomial incidences [5]. Like the rest of *S. aureus* species, MRSA strains are biologically enhanced for prolonged survival on dry surfaces, while the magnitude of contamination can generally be influenced by compliance to hygienic measures [6, 7].

On the other hand, touch frequency, patient load, population of MRSA colonized/infected patients, length of hospital stays, and invasive procedures like catheterization are also known to predict for contamination rates [8, 9]. High touch surfaces and items in the immediate vicinity of patients such as bed surfaces, floor, linen, sink hampers, door handles are reported to be more frequently and heavily contaminated [10–12]. Air, especially in controlled environments such as operating theatres and tape water have also been associated with spread of multi-drug resistant (MDR) pathogens in several hospital settings [13].

Despite the anticipated important role of hospital environment in transmission of MRSA, less emphasis has been given in evaluating the occurrence of these pathogens in our hospital settings. Generally, few studies have adequately assessed the relative role of the environment versus other modes of transmission of hospital-acquired pathogens. Bacteriological sampling of environmental surfaces has been only indicated as part of some outbreak investigations but rarely in endemic situations. Identifying the magnitude and potential environmental sites for MRSA contamination could contribute towards emphasis on various hygienic measures hence reduced cross transmissions and subsequent HAI’s.

This study was undertaken to assess MRSA contamination of inanimate surfaces surrounding patients receiving care at Muhimbili National Hospital (MNH), the largest tertiary hospital in Tanzania.

## Main text

### Methods

#### Study design and study area

This cross-sectional study was conducted at MNH in Dar es Salaam, Tanzania between May and June 2017. MNH is the largest tertiary healthcare facility admitting up to 1200 inpatients per week with nearly 3000 employees, also receiving a minimum of 2000 patient’s visitors per day; population size and complexity for possible contamination of “high touch” surfaces. Specimens were from surfaces of selected high touch items in general wards, intensive care units (ICUs) and operating rooms and taken to Muhimbili University of Health and Allied Sciences (MUHAS) Microbiology laboratory for processing.

#### Specimen collection and MRSA detection

**Fig. 1:**
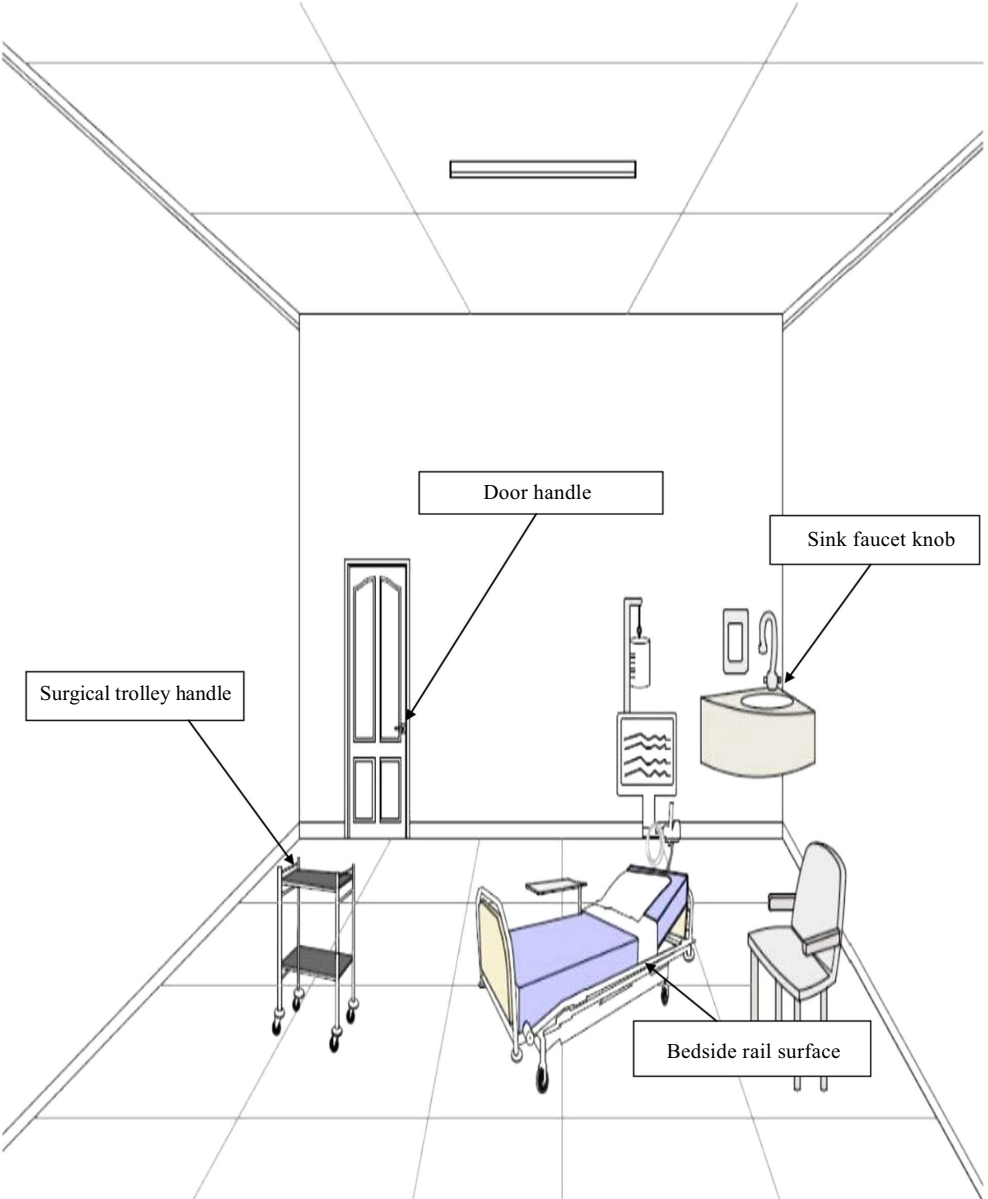
Schematic of the locations of sample collection from the selected sources

Sterile moistened swabs were used to collect specimens from surfaces of highly touched items (bed surfaces, surgical trolleys, door knobs and sinks), which were conveniently chosen from studied patient’s rooms (Fig. 1).

All specimens were collected 1 h after daily cleaning and disinfection with the aim of deriving an overview on effectiveness of disinfection process, therefore the need to control for exposure length of the sampled items.

Surfaces were sampled based on recommendations of the Centers for Disease Control and Prevention (CDC) environmental cleaning toolkit [14] whereby specimens were collected using sterile cotton swabs by gently rubbing the swab moistened with sterile physiological saline on the surfaces and rotating the swab round to 360°. A standardized surface area (of not greater than 10 cm^2^) was swabbed for each selected item.

All swabs were pooled in tubes containing Amies transport media. Tubes were labeled for specimen sources, date and time of collection, the hospital unit and immediately delivered to MUHAS Microbiology laboratory for processing.

Swabs specimens were immediately cultured on plates containing MRSA screening agar (Liofilchem™ Italy) and incubated aerobically at 33–35 °C for 24 or 24 h more if no growth, after which any growth with colony color ranging from mauve-red-pink was indicative for MRSA (Liofilchem™ Italy). Further identification of *S. aureus* was done using conventional bacteriological tests (including gram staining, catalase and coagulase tests).

Kirby Bauer disk diffusion using cefoxitin disk was a method for confirmation of MRSA according to the guidelines of the Clinical and Laboratory Standards Institute (CLSI 2013) [15].

Colony suspension equivalents to 0.5 Macfarland solutions were inoculated on Mueller–Hinton Agar (Liofilchem™ Italy). Cefoxitin disks (30 μg, Liofilchem™ Italy) were placed onto media and incubated aerobically at 35 °C for 24 h. All isolates resistant to Cefoxitin were considered as MRSA, a growth inhibition zone of 21 mm or less around Cefoxitin disk indicated MRSA [15]. *S. aureus* (ATCC 25923 and ATCC 29213) were used for quality control.

#### Data analysis

Data obtained were analyzed using Statistical Package for Social Sciences (SPSS) version 20.0. Descriptive statistics; frequencies and cross tabulation were used, binary logistic regression was used to obtain the odds ratio for the significant predictors. A *p* value of less than 0.05 was considered to be statistically significant.

### Results

Fifty patients’ care rooms comprising of 36 general wards, seven ICU’s and seven operating rooms were included in the study as sources of sample. Of the patients’ rooms studied, 15 were occupied by males and 19 by female while 16 were used by both genders including ICU’s and operating rooms. Thirty-one rooms accommodated 10 or more patients while 12 rooms had less than 10 patients. The hospital cleanness and disinfection protocol in these facilities involved the use of diluted commercial disinfectant (diluted Sodium hypochlorite solution), liquid soaps and mops to clean various items surrounding patients receiving care. Cleanness and disinfection was done twice a day at 5 h intervals, no special protocol was in place for objects considered highly touched.

#### Contamination and distribution of MRSA

A total of 200 environmental samples were collected, of which 108 samples gave no growth on MRSA selective agar; 40 specimens had growth features not distinctive for MRSA; 11 isolates were negative for *S. aureus* identification tests and 41 samples grew *S. aureus.* Among *S. aureus* isolates, 39 were confirmed to be MRSA using Cefoxitin disk making a prevalence of 19.5% (39/200).

Amongst the hospital units, general wards presented with most contaminated items with no statistically significance difference between those in surgical wards (21.0%) and medical wards (20.5%), *p *= 0.899. Seventeen percent (17.9%) of items from ICU’s had MRSA contamination where as the items from operating theatres presented with the least contamination (14.3%) (Table 1).

**Table 1:** Contamination and distribution of MRSA in various units/wards and studied surfaces

Variables	Total specimens	Positive (%)	*p*-value
Overall magnitude	200	39 (19.5)	
General wards			
Medical	44	9 (20.5)	0.899
Surgical	100	21 (21.0)	
Special units			
Intensive care unit	28	5 (17.9)	0.812
Operating theater	28	4 (14.3)	
Gender of occupants			
Female’s units	76	22 (28.9)	0.026
Male’s units	60	8 (13)	
Number of patients			
≥ 10 patients	76	23 (30.3)	0.006
< 10 patients	96	12 (12.5)	
Surfaces			
Bed surfaces	50	17 (34)	0.010
Door handles	50	13 (26)	
Sinks	50	6 (12)	
Surgical trolleys	50	3 (6)	

MRSA contamination on surfaces of items found in areas occupied by female patients was significantly higher (28.9%) than that of items found in male’s patients’ areas (13%), (*p *= 0.026). As for studied items the highest contamination was seen in bed surfaces (34%), while surgical trolleys were least contaminated (6%) (Table 1).

#### Correlate factors for environmental MRSA contamination

Variables that showed significant association by Chi square independence tests; patients’ number, gender of room occupants and specimen source were further analyzed for binary logistic regression model to determine their odds ratios. Variables that remained significantly associated with environmental contamination by the bivariate model were: ten or more patients in a room (odds ratio [OR] 4.75 [95% confidence interval (CI) 1.624–13.895]; *p* = 0.004), Sources of the specimen; Bed surfaces (OR 6.26 [95% CI 1.443–27.153]; *p* = 0.014), and door handles (OR 5.21 [95% CI 1.321–25.426]; *p* = 0.036) (Table 2). Female’s occupant was more likely to be contaminated with MRSA strain compared to males occupant (OR 1.650 [95% CI 0.139–19.571]; *p* = 0.691).

**Table 2:** Correlates of MRSA contamination by bivariate logistic analysis

Variables (number of specimens)	MRSA (%)	OR (95% CI)	*p*-value
Gender of occupants			
Female (76)	22 (28.9)	1.650 (0.139–19.571)	0.691
Males (60)	7 (11.7)	1	
Number of patients			
≥ 10 patients (76)	23 (30.3)	4.75 (1.624–13.895)	0.004
< 10 patients (96)	12 (12.5)	1	
Specimen source			
Bed surfaces (50)	17 (34)	6.26 (1.443–27.153)	0.014
Door handles (50)	13 (26)	5.21 (1.321–25.426)	0.036
Sinks (50)	6 (12)	0.906 (0.296–2.771)	0.863
Surgical trolleys (50)	3 (6)	1	

### Discussion

To the best of our knowledge, this was the first study in our settings undertaken to elucidate the role of frequently accessed items in patient’s care environment as secondary reservoir of medical important pathogens. Of important findings is the high contamination of MRSA among studied surfaces (19.5%) which has similarly been reported from studies in Uganda (19%) and Egypt (21.8%) and higher compared to findings from a studies in Nigeria (5%) and Ethiopia (1.7%) [12, 16–18]. Variations in the detection rate of MRSA in hospital environment have been reported in some studies possibly due to differences in patient colonization load, hospital’s cleaning/disinfection protocols, study design including timing of sample collection and laboratory method used [6–9]. Nevertheless, as in this study samples were taken shortly after daily cleaning, our findings provide an alarming indication on ineffectiveness of the process.

High MRSA contamination of items from general wards compared to ICU’s and operating rooms is in keeping with reports from studies done in Egypt and Nigeria [16, 18]. The difference in the findings can be explained by the minimized number of patients and personnel flow as well as increased adherence to hand hygiene in the later settings. However measures to prevent cross transmission should be warranted to ICU’s and operating rooms even with the anticipated low levels of MRSA, owing to compromised patients attended therein.

We have also reported bed surfaces as highly MRSA contaminated (34%) amongst studied items, findings in line with reports in Nigeria and United Kingdom where in both studies bed surfaces took the lead on the contaminated objects [15, 19]. The beds surfaces are considered patient contact surfaces and therefore the detected pathogens might have been shed by the infected/colonized patients occupying the particular beds.

Patient’s load as factor for contamination has also been implicated elsewhere, in which fewer patient bedrooms design had significantly reduced HAI’s compared to semi private or open wards design [19]. Generally number of patients in a unit would reflect poor hygiene compliance and/quicker recontamination rates from colonized visitors or health care workers (HCW’s) even in an event of effective decontamination process.

Higher prevalence of MRSA in facilities with female patients’ documented in this study portrays the contrary nature of association between contamination and patients’ gender as the findings from elsewhere reported lesser contamination in female wards [9]. The role of gender in defining the contamination rates in hospital settings can be connected to difference in hygiene practices or the different rate of MRSA colonization/infection between male and females in the hospital, both of which were not measured in this study.

### Conclusions

Areas of hospital environment presents underestimated important reservoir for HAI’s associated pathogens even in non-outbreak settings where by patients load and nature of sites can be important predictors. Routine surveillance of hospital environment contamination and larger prospective studies are warranted to assess the correlation between environmental MRSA and the acquisition of MRSA by patients or the vice versa.

## Limitations

We didn’t asses the individual variability among the cleaners on compliance to the disinfection protocol, neither was MRSA colonization and/infection among patients and HCW’s, both of which could have influenced the MRSA detection rates.

## Abbreviations

ATCC: American type culture collection
CDC: Centers for Disease Control and Prevention
CLSI: Clinical and laboratory standards institute guidelines
HCW: health care worker
MRSA: methicillin resistant *Staphylococcus aureus*
MUHAS: Muhimbili University of Health and Allied Sciences
